# Supplementary material for: Insights on pathoadaptation of sequential Pseudomonas aeruginosa isolates to the urinary tract
Source: Microbiol Spectr. 2025 Jul 29;13(9):e00456-25. doi: 10.1128/spectrum.00456-25 (PMC12403851; doi:10.1128/spectrum.00456-25)
Supplement: Supplemental material — Tables S1 to S6; Fig. S1 to S3. [file spectrum.00456-25-s0001.docx]

**SUPPLEMENTAL MATERIAL**

**LEGENDS**

**Table S1** Mean CFU/mL counts and final OD_600_ of early and late isolates from patients A, D and F after 24h of culture in TS.

**Table S2** Generation time (min) of early and late isolates from patients A (A), D (B) and F (C) grown in TS, HU and AUM.

**Table S3** Generation time (min) of early and late isolates from patients A (A), D (B) and F (C) grown in TS with different NaCl concentrations (0 M, 0.1 M, 0.2 M, 0.3 M, 0.4 M).

**Table S4** Generation time (min) of early and late isolates from patients A (A), D (B) and F (C) grown in TS at pH 7, 6, 5.5 and 5.

**Table S5** Generation time of early and late isolates from patients A (A), D (B) and F (C) grown in TS under different H_2_O_2_ concentrations (0 mM, 0.1 mM, 0.2 mM, 0.3 mM and 0.4 mM).

**Table S6** Percentage survival of *G. mellonella* larvae after 48H injection of early and late isolates from patients A, D and F.

**Table S7** List of proteins significantly overabundant and underabundant of growing cell (OD of 1) of *P. aeruginosa* strains grown in TS, AUM and HU.

**FIG S1** Principal component analysis (PCA) of top 500 variable proteins (A) and PCA of all proteins according to the medium (B) from proteomic analysis of the six *P. aeruginosa* isolates (one early and one late isolates per patient).

**FIG S2** Venn diagrams representing the number of metabolic categories of overabundant proteins in TS (1) or HU (2) media common or not to early (A) or late (B) isolates of the three patients A, D and F.

**FIG S3** Representative pictures of swimming and swarming tests of early and late isolates from patients A, D and F and the PA14 reference strain used as a positive control.

**Table S1**

| **Counts (CFU/mL)** | **A-e** | **A-l** | **D-e** | **D-l** | **F-e** | **F-l** |
| --- | --- | --- | --- | --- | --- | --- |
| **Mean** | 7,00E+08 | 8,55E+08 | 5,90E+08 | 4,40E+08 | 5,00E+08 | 7,00E+08 |
|  | (final OD = 1.68) | (final OD = 1.8) | (final OD = 1.6) | (final OD = 0.86) | (final OD = 1.76) | (final OD = 1.5) |

**Table S2**

| **Generation time (min)** | **A-e** | **A-l** | **D-e** | **D-l** | **F-e** | **F-l** |
| --- | --- | --- | --- | --- | --- | --- |
| **TS** | 33,33 | 31,31 | 24,96 | 41,42 | 26,5 | 24,37 |
|  | 34,92 | 33,21 | 28,83 | 50,92 | 25,52 | 35,8 |
|  | 20,49 | 23,9 | 20,89 | 54,95 | 26,98 | 28,99 |
|  | 38,49 | 33,9 | 27,42 | 52,13 | 28,75 | 34,94 |
|  | 34,52 | 33,52 | 27,31 | 52,54 | 28,48 | 31,76 |
|  | 32,13 | 34,25 | 20,15 | 36,67 | 28,61 | 32,33 |
|  | 31,4 | 33,58 | 26,21 | 53,23 | 26,29 | 31,87 |
|  | 32,94 | 33,49 | 17,44 | 39,71 | 27,08 | 29,23 |
|  | 29,25 | 31,85 | 26,88 | 51,47 | 28,34 | 34,35 |
|  | 36,45 | 36,32 | 22,9 | 40,29 | 27,09 | 35,9 |
|  | 34,63 | 36,77 | 27,3 | 45,99 | 28,03 | 31,22 |
|  | 36 | 37,14 | 28,8 | 51,78 | 28,21 | 34,55 |
|  | 37,82 | 35,1 | 26,59 | 48,34 |  |  |
| **HU** | 55,32 | 40,67 | 24,17 | 87,1 | 34,81 | 40,63 |
|  | 52,17 | 39,27 | 25,26 | 68,73 | 32,35 | 41,52 |
|  | 52,75 | 39,79 | 26,26 | 51,45 | 33,15 | 38,94 |
|  |  |  | 14,94 | 53,05 |  |  |
| **AUM** | 45,01 | 26,3 | 32,42 | 71,47 | 33,82 | 51,92 |
|  | 43,39 | 31,26 | 31,72 | 74,55 | 32,88 | 51,83 |
|  | 42,06 | 34,12 | 36,88 | 70,92 | 33,64 | 47,2 |
|  |  |  | 33,36 | 68,43 |  |  |

**Table S3**

| **Generation time (min)** | **A-e** | **A-l** | **D-e** | **D-l** | **F-e** | **F-l** |
| --- | --- | --- | --- | --- | --- | --- |
| **0 M** | 33,33 | 31,31 | 24,96 | 41,42 | 26,5 | 24,37 |
|  | 34,92 | 33,21 | 28,83 | 50,92 | 25,52 | 35,8 |
|  | 20,49 | 23,9 | 20,89 | 54,95 | 26,98 | 28,99 |
|  | 38,49 | 33,9 | 27,42 | 52,13 | 28,75 | 34,94 |
|  | 34,52 | 33,52 | 27,31 | 52,54 | 28,48 | 31,76 |
|  | 32,13 | 34,25 | 20,15 | 36,67 | 28,61 | 32,33 |
|  | 31,4 | 33,58 | 26,21 | 53,23 | 26,29 | 31,87 |
|  | 32,94 | 33,49 | 17,44 | 39,71 | 27,08 | 29,23 |
|  | 29,25 | 31,85 | 26,88 | 51,47 | 28,34 | 34,35 |
|  | 36,45 | 36,32 | 22,9 | 40,29 | 27,09 | 35,9 |
|  | 34,63 | 36,77 | 27,3 | 45,99 | 28,03 | 31,22 |
|  | 36 | 37,14 | 28,8 | 51,78 | 28,21 | 34,55 |
|  | 37,82 | 35,1 | 26,59 | 48,34 |  |  |
| **0.1 M** | 34,31 | 37,56 | 21,36 | 33,39 | 27,11 | 36,04 |
|  | 33,15 | 50,19 | 19,62 | 47,78 | 28,57 | 35,47 |
|  | 33,39 | 35,08 | 25,87 | 53,51 | 28,22 | 34,26 |
| **0.2 M** | 36,26 | 47,46 | 27,84 | 52,52 | 27,77 | 27,06 |
|  | 32,62 | 49,62 | 28,03 | 47,78 | 31,39 | 40,13 |
|  | 32,56 | 42,71 | 28,71 | 55,41 | 31,9 | 41,17 |
| **0.3 M** | 37,13 | 33,63 | 30,19 | 60,19 | 34,02 | 49,67 |
|  | 35,45 | 42,22 | 26,67 | 60,48 | 34,15 | 44,09 |
|  | 31,97 | 28,06 | 28,27 | 60,36 | 33,97 | 47,03 |
| **0.4 M** | 40,75 | 34,23 | 28,55 | 62,11 | 36,64 | 60,41 |
|  | 37,24 | 47,28 | 29,73 | 71,58 | 39,09 | 52,15 |
|  | 36,01 | 38,46 | 22,36 | 66,5 | 37,44 | 51,31 |

**Table S4**

| **Generation time (min)** | **A-e** | **A-l** | **D-e** | **D-l** | **F-e** | **F-l** |
| --- | --- | --- | --- | --- | --- | --- |
| **pH 7** | 33,33 | 31,31 | 24,96 | 41,42 | 26,5 | 24,37 |
|  | 34,92 | 33,21 | 28,83 | 50,92 | 25,52 | 35,8 |
|  | 20,49 | 23,9 | 20,89 | 54,95 | 26,98 | 28,99 |
|  | 38,49 | 33,9 | 27,42 | 52,13 | 28,75 | 34,94 |
|  | 34,52 | 33,52 | 27,31 | 52,54 | 28,48 | 31,76 |
|  | 32,13 | 34,25 | 20,15 | 36,67 | 28,61 | 32,33 |
|  | 31,4 | 33,58 | 26,21 | 53,23 | 26,29 | 31,87 |
|  | 32,94 | 33,49 | 17,44 | 39,71 | 27,08 | 29,23 |
|  | 29,25 | 31,85 | 26,88 | 51,47 | 28,34 | 34,35 |
|  | 36,45 | 36,32 | 22,9 | 40,29 | 27,09 | 35,9 |
|  | 34,63 | 36,77 | 27,3 | 45,99 | 28,03 | 31,22 |
|  | 36 | 37,14 | 28,8 | 51,78 | 28,21 | 34,55 |
|  | 37,82 | 35,1 | 26,59 | 48,34 |  |  |
| **pH 6** | 28,85 | 37,5 | 23,45 | 50,26 | 26,33 | 27,61 |
|  | 27,43 | 35,62 | 23,04 | 53,35 | 25,95 | 25,8 |
|  | 24,72 | 38,72 | 24,6 | 53,58 | 27,91 | 34,83 |
| **pH 5.5** | 32,63 | 27,75 | 23,9 | 49,19 | 23,94 | 33,94 |
|  | 37,45 | 31,02 | 23,39 | 51,7 | 21,65 | 31,1 |
|  | 35,3 | 29,41 | 24,16 | 52,65 | 25,34 | 34,87 |
| **pH 5** | 42,43 | 48,62 | 27,48 | 51,6 | 28,06 | 40,71 |
|  | 45,6 | 45,28 | 25,91 | 57,81 | 28,24 | 41,82 |
|  | 42,61 | 39,93 | 30 | 58,92 | 29,66 | 48,56 |

**Table S5**

| **Generation time (min)** | **A-e** | **A-l** | **D-e** | **D-l** | **F-e** | **F-l** |
| --- | --- | --- | --- | --- | --- | --- |
| **0 mM** | 33,33 | 31,31 | 24,96 | 41,42 | 26,5 | 24,37 |
|  | 34,92 | 33,21 | 28,83 | 50,92 | 25,52 | 35,8 |
|  | 20,49 | 23,9 | 20,89 | 54,95 | 26,98 | 28,99 |
|  | 38,49 | 33,9 | 27,42 | 52,13 | 28,75 | 34,94 |
|  | 34,52 | 33,52 | 27,31 | 52,54 | 28,48 | 31,76 |
|  | 32,13 | 34,25 | 20,15 | 36,67 | 28,61 | 32,33 |
|  | 31,4 | 33,58 | 26,21 | 53,23 | 26,29 | 31,87 |
|  | 32,94 | 33,49 | 17,44 | 39,71 | 27,08 | 29,23 |
|  | 29,25 | 31,85 | 26,88 | 51,47 | 28,34 | 34,35 |
|  | 36,45 | 36,32 | 22,9 | 40,29 | 27,09 | 35,9 |
|  | 34,63 | 36,77 | 27,3 | 45,99 | 28,03 | 31,22 |
|  | 36 | 37,14 | 28,8 | 51,78 | 28,21 | 34,55 |
|  | 37,82 | 35,1 | 26,59 | 48,34 |  |  |
| **0.1 mM** | 32,15 | 20,62 | 27,81 | 55,42 | 27,53 | 34,6 |
|  | 35,12 | 35,86 | 28,73 | 55,37 | 27,81 | 33,61 |
|  | 34,72 | 36,7 | 27,29 | 47,29 | 28,48 | 34,95 |
|  | 34,71 | 36,28 |  |  | 28,81 | 32,37 |
| **0.2 mM** | 36,11 | 35,76 | 27,83 | 50,61 | 28,86 | 34,86 |
|  | 34,12 | 35,45 | 28,41 | 50,17 | 27,85 | 29,74 |
|  | 34,29 | 34,69 | 27,31 | 52,73 | 28,92 | 34,12 |
|  | 33,5 | 33,68 |  |  | 26,72 | 32,14 |
| **0.3 mM** | 34,47 | 35,61 | 28,83 | 51,46 | 26,44 | 31,12 |
|  | 35,04 | 35,01 | 28,81 | 48,58 | 27,91 | 28,82 |
|  | 36,32 | 32,16 | 29 | 51,12 | 28,84 | 35,55 |
|  | 37,38 | 34,71 |  |  | 27,52 | 42,49 |
| **0.4 mM** | 35,8 | 36,14 | 29,6 | 52,71 | 29,07 | 37,38 |
|  | 34,67 | 34,6 | 29,91 | 56,16 | 28,63 | 34,54 |
|  | 35,45 | 36,61 | 29,99 | 41,61 | 29,81 | 36,37 |
|  | 36,87 | 37,45 |  |  | 26,88 | 29,6 |

**Table S6**

| **% of surviving larvae** | **A-e** | **A-l** | **D-e** | **D-l** | **F-e** | **F-l** |
| --- | --- | --- | --- | --- | --- | --- |
| **48H post-infection** | 0 | 100 | 40 | 100 | 100 | 60 |
|  | 60 | 100 | 50 | 100 | 0 | 100 |
|  | 40 | 100 | 60 | 100 | 50 | 100 |
|  | 40 | 100 |  | 100 | 10 | 100 |


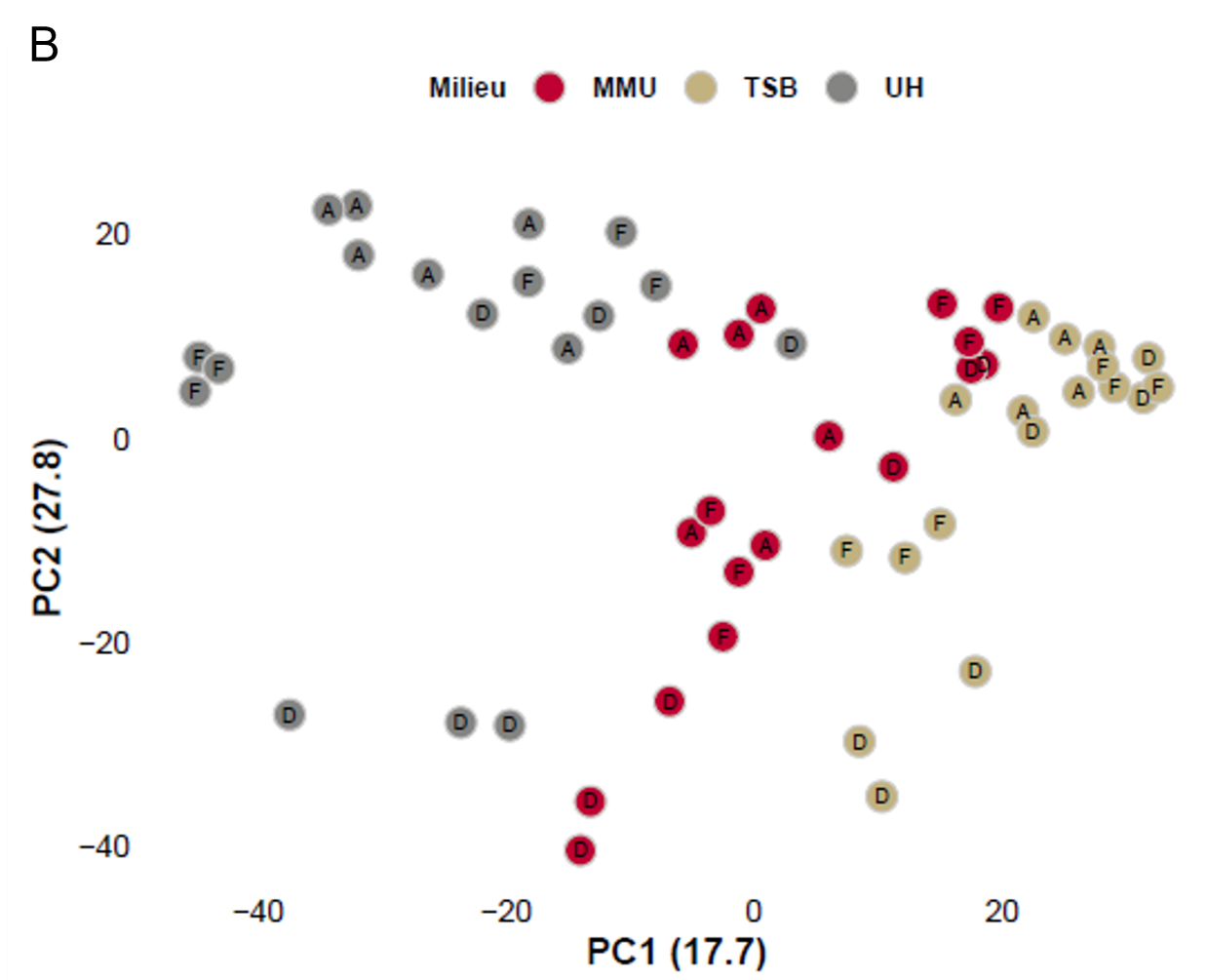
**Figure S1**


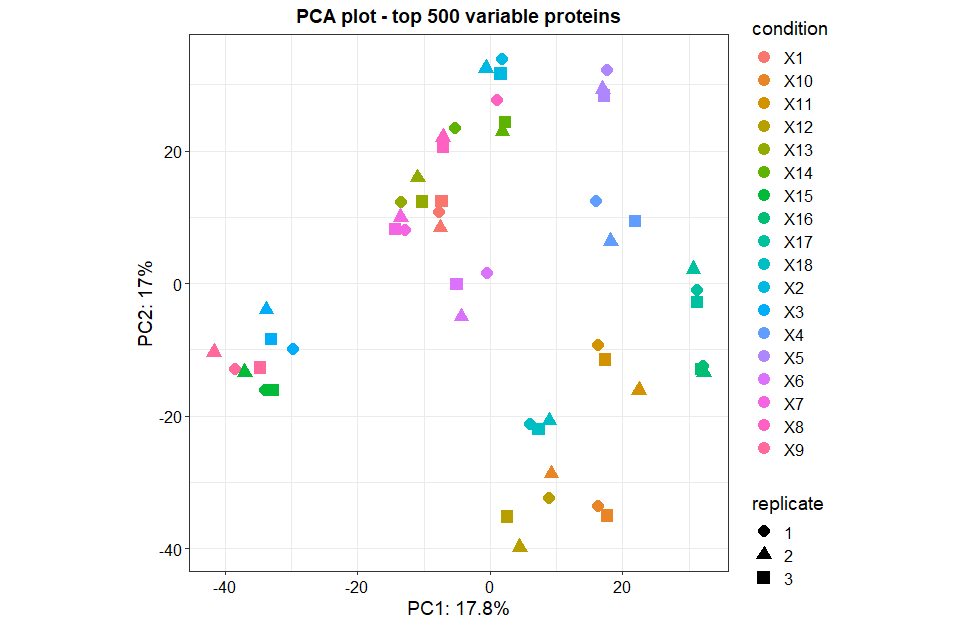


A

**
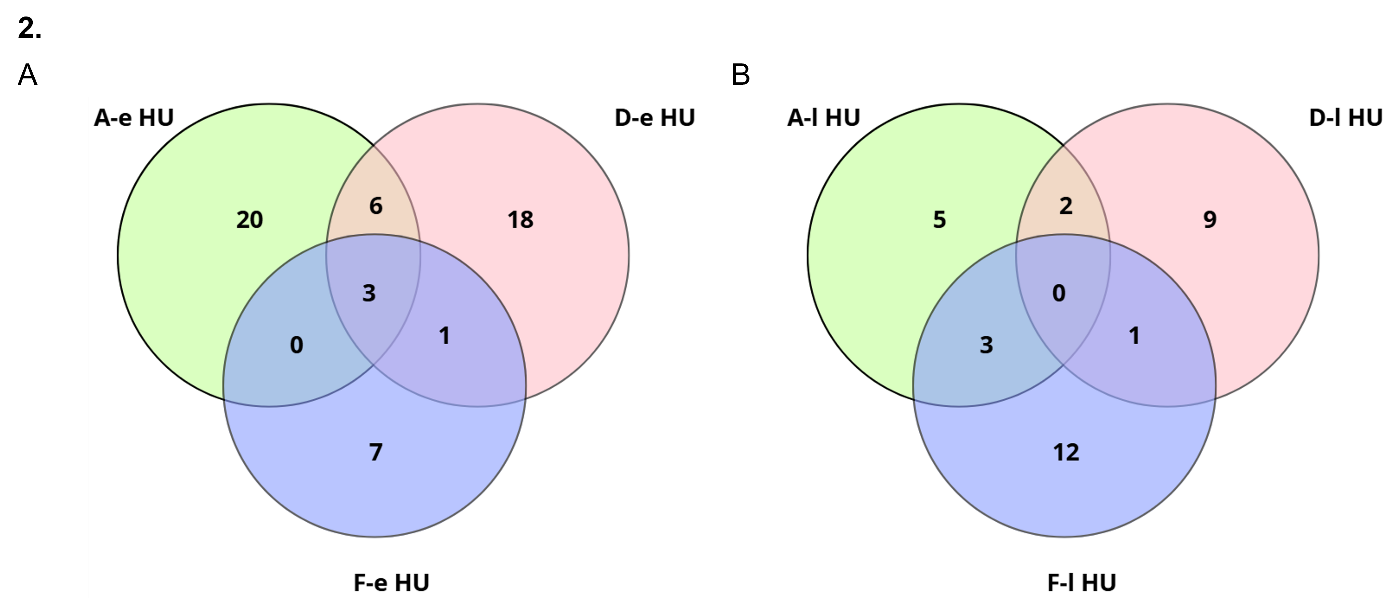

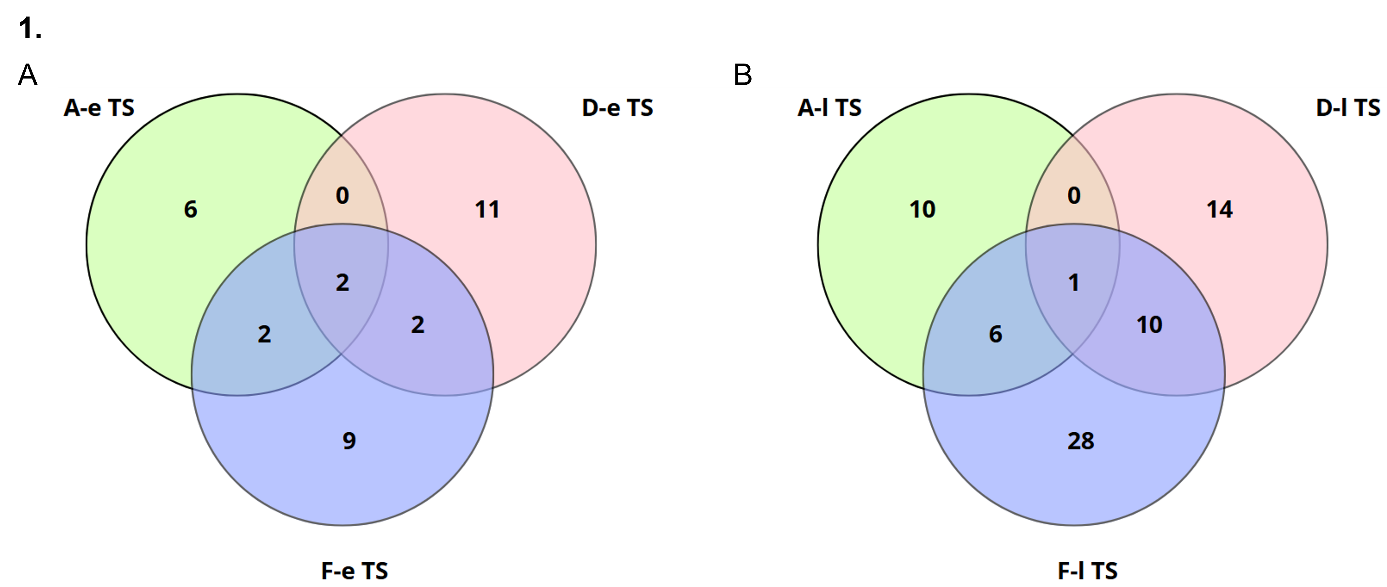
Figure S2**


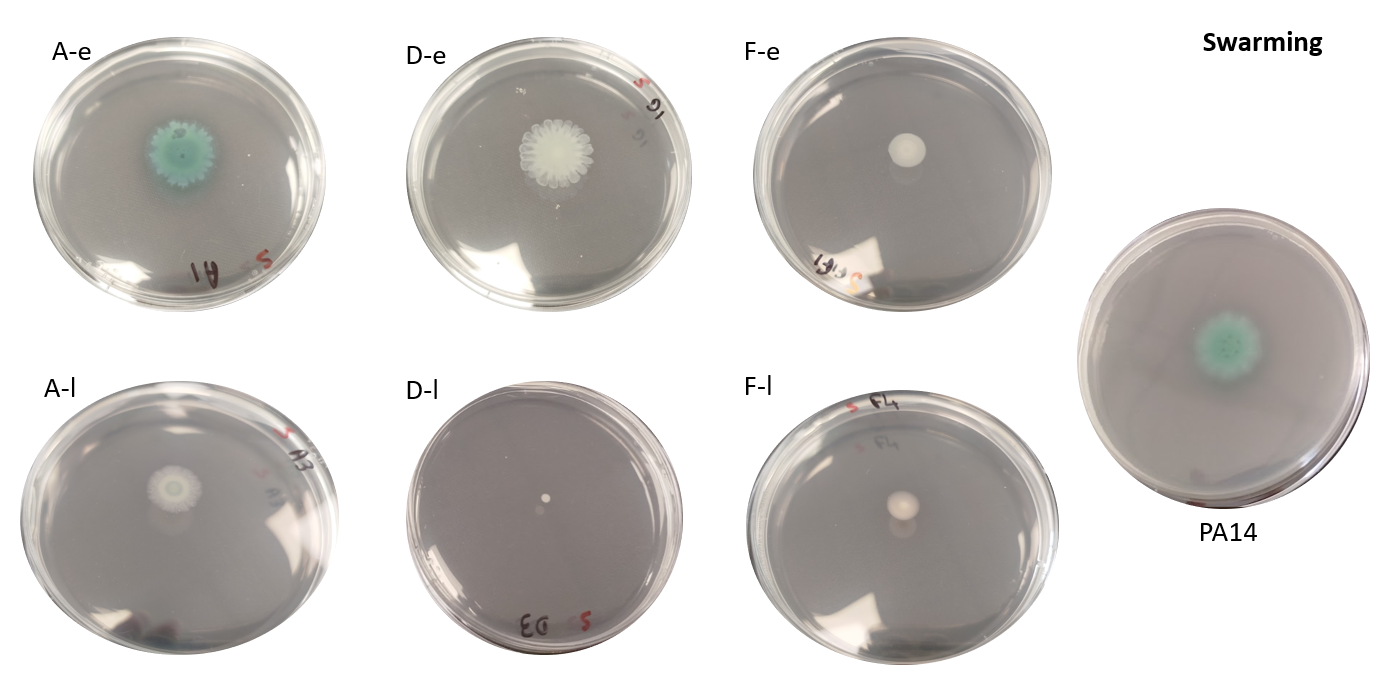

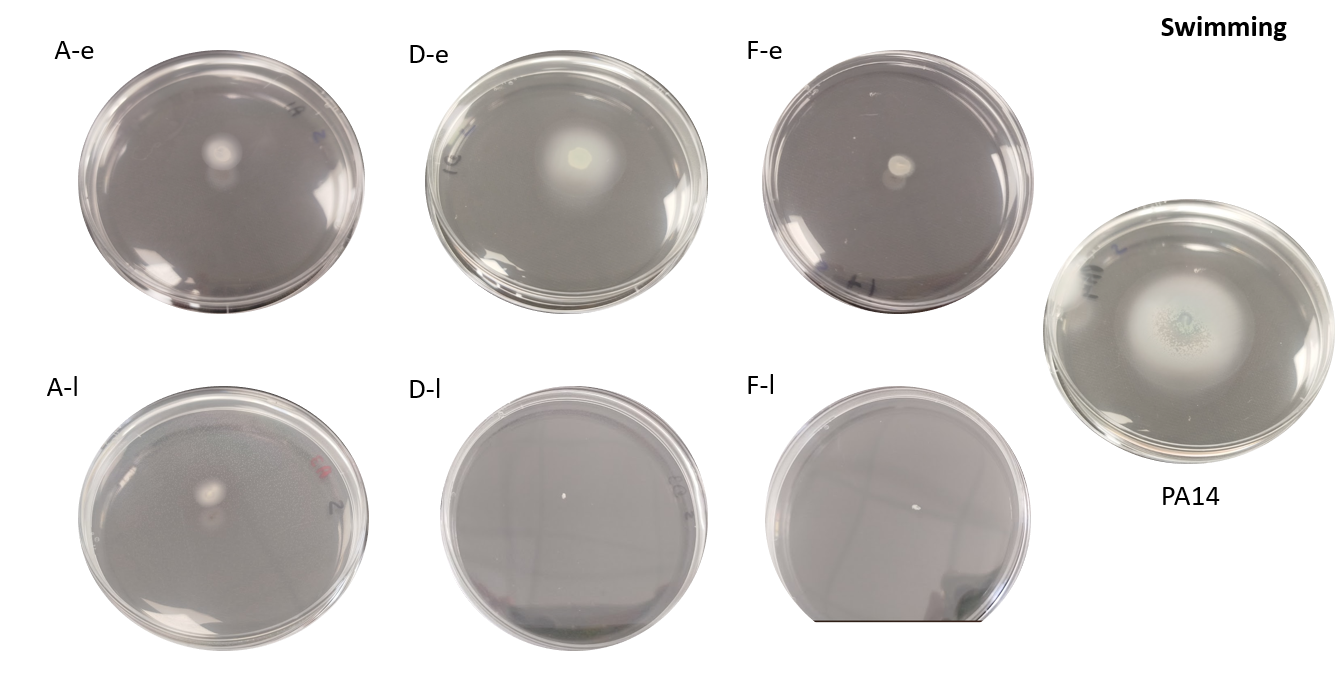


**Figure S3**
